# The effect of influenza vaccine in reducing the severity of clinical outcomes in patients with COVID-19: a systematic review and meta-analysis

**DOI:** 10.1038/s41598-022-18618-6

**Published:** 2022-08-22

**Authors:** Hossam Waleed Almadhoon, Aboalmagd Hamdallah, Sarah Makram Elsayed, Abdulrahman Ibrahim Hagrass, Mohammed Tarek Hasan, Aya Mamdouh Fayoud, Mohammed Al-kafarna, Mohammad Elbahnasawy, Fadel Alqatati, Khaled Mohamed Ragab, Mohamed Sayed Zaazouee, Elfatih A. Hasabo

**Affiliations:** 1 Faculty of Dentistry, Al-Azhar University - Gaza, Gaza Strip, Palestine; 2 Faculty of Medicine, Al-Azhar University, Damietta, Egypt; 3 Faculty of Medicine, October 6 University, Giza, Egypt; 4 Faculty of Medicine for Boys, Al-Azhar University, Cairo, Egypt; 5 Faculty of Pharmacy, Kafr El Sheikh University, Kafr El Sheikh, Egypt; 6 Faculty of Pharmacy, Al-Azhar University - Gaza, Gaza Strip, Palestine; 7 Faculty of Medicine, Alexandria University, Alexandria, Egypt; 8 Faculty of Medicine, Minia University, Minia, Egypt; 9 Faculty of Medicine, Al-Azhar University, Assiut, Egypt; 10 Faculty of Medicine, University of Khartoum, Khartoum, Sudan; 11 International Medical Research Association (IMedRA), Cairo, Egypt

**Keywords:** Diseases, Infectious diseases

## Abstract

Recent evidence suggests that vaccination against influenza may reduce the clinical outcomes of COVID-19. This study looked at the link between influenza vaccination and the severity of COVID-19 infection. We searched five databases until August 2021. We included studies that reported the relationship between influenza vaccination and COVID-19 outcomes. We pooled the data as risk ratio (RR) or mean difference (MD), with 95% confidence intervals (CIs), the data pooled using fixed and random effects models according to the heterogeneity of results. Sixteen observational studies with 191,496 COVID-19 patients were included. In terms of mechanical ventilation, our analysis showed a significant favor for the influenza vaccinated group over the non-vaccinated group (RR = 0.72, 95% CI [0.54, 0.96], *P* = 0.03). However, the analysis indicated no statistically significant differences between vaccinated and non-vaccinated groups in the term of mortality rate (RR = 1.20, 95% CI [0.71, 2.04], *P* = 0.50), hospital admissions (RR = 1.04, 95% CI [0.84, 1.29], *P* = 0.75), intensive care admissions (RR = 0.84, 95% CI [0.44, 1.62], *P* = 0.60). There were no significant differences between those who had received the influenza vaccine and those who had not in COVID-19 clinical outcomes, except for mechanical ventilation which showed a significantly lower risk in the influenza vaccinated group compared to the non-vaccinated one. However, future research is encouraged as our data have limitations, and the influenza vaccine is regularly updated. Also, this does not exclude the importance of the influenza vaccine during the COVID-19 pandemic.

## Introduction

The World Health Organization (WHO) announced the pandemic of coronavirus disease 2019 (COVID-19) in March 2020^1^. COVID-19 is a respiratory syndrome with a wide range of severity that is caused by severe acute respiratory syndrome coronavirus 2 (SARS-CoV-2)^2^. COVID-19 pandemic has invaded the world, leading to global physical and mental health effects^3^. The pandemic's severity emphasizes how crucial it is to administrate FDA-authorized vaccines successfully. On the other hand, vaccine hesitancy can be considered a significant hazard towards achieving this^4^.

Meanwhile, influenza also represents a considerable public health burden despite the presented therapeutic and preventative measures^5^. Vaccines against influenza have been used for over 60 years. There have been evolved and improved methods to evaluate the vaccine efficacy and effectiveness in that time; however, there are still challenges. Those include influenza vaccine effectiveness being a moving target. Influenza viruses keep evolving, so vaccine strains are regularly updated, the population's immune profile changes and novel vaccine products are being developed to provide improved protection^6^.

According to the WHO and the Pan American Health Organization (PAHO), COVID-19 and influenza viruses share similarities in terms of disease presentation and modes of transmission^7^. However, there are some differences, for example, the speed of transmission, as influenza has a shorter incubation period as well as serial interval compared to COVID-19. The influenza virus's serial interval is three days, but, that for COVID-19 was estimated as five to six days, indicating that influenza could spread faster than COVID-19^7^. On the hand, the Centers for Disease Control and Prevention (CDC) recently stated that, generally, COVID-19 virus is more contagious than influenza viruses, and that COVID-19 can spread, in a quick and easy way, to plenty of people. Overall, resulting in a continual spreading among people as time progresses^8^. Another difference includes that COVID-19 appeared to cause more severe illnesses, and that COVID-19 serious illness could lead to hospitalization and mortality even in healthy individuals^8^.

The suggested theoretical mechanisms for a potential protective effect of the influenza vaccine against COVID-19 included the MF59 presence in the influenza vaccine, which was shown to help in potentiating an immune response against SARS-CoV variants^9^. Another proposal included if the vaccine could stimulate enough trained innate immune memory; so, that when another respiratory pathogen such as SARS‐CoV‐2 occurred, the local lung immune system would be primed for a rapid response^10^. Overall, this could affect the SARS‐Cov‐2 acquisition or the COVID‐19 disease course^10^. Furthermore, another theory was based on the suggestion that influenza and COVID-19 viruses engage with the angiotensin-converting enzyme 2 (ACE-2) and tetraspanin antibodies. Therefore, ACE-2 and tetraspanin antibodies might inhibit covid-19 and low-pathogenic influenza A virus^11^.

There have been controversial results about whether there is an association between influenza vaccination and the severity of COVID-19 outcomes or not^12,13^. Therefore, this study aims to solve the controversial results by investigating whether influenza vaccination reduces the severity of COVID-19 in patients or not.

## Methods

### Protocol and registration

This review follows the recent updates of PRISMA-P^14^, and MOOSE^15^ guidelines. Our protocol was a priori registered in the PROSPERO registry (CRD42021273299).

### Literature search

We conducted our searches in the following databases; Embase, PubMed, Scopus, Web of Science, OVID, and Cochrane Central database, till the 5th of August 2021. We used the following search strategy for our search on PubMed: ((Flu OR Influenza OR Influenza virus) AND (“COVID 19” OR COVID-19 OR SARS-CoV-2 OR SARS2 OR 2019-nCoV OR Coronavirus OR Corona OR “Coronavirus Disease 2019” OR “Coronavirus Disease-19” OR “Novel Coronavirus”)) AND (vaccination* OR vaccine* OR shot OR jabs). Then we adapted to other databases: We also conducted a manual search including the references of our including studies and the google scholar search.

### Selection criteria

Our criteria include observational retrospective or prospective studies (case–control, and cohort studies) of living human beings that met the following criteria: (1) patients were diagnosed with COVID-19; (2) studies that compared COVID-19 patients who received influenza vaccine against patients who did not receive it. We excluded (1) any study that did not report health-related outcomes; (2) studies that did not use influenza vaccine as exposure; (3) studies that did not identify the status of patients if they are COVID-19 or not; (4) ecological studies; (5) duplicated studies or studies with untrusted data; (6) studies written in a language other than English; (7) animal studies, conference abstract, book chapter, letter, editorial, comment.

Two independent reviewers (H.W.A. and A.H.) screened the titles and abstracts of retrieved records; then they screened the eligibility of studies to our criteria by full-text screening. They classified them into included, excluded, or undecided studies. We solved disagreements by consulting a third reviewer (M.S.Z) whenever necessary.

### Quality assessment

We evaluated our included studies using the Newcastle–Ottawa Quality Assessment Scale for case–control or cohort studies^16^. This scale consists of a star-awarding system for each specific methodological section, and according to this system, an overall quality score was generated for each study. The scale ranges from 0 to 9 points, where the studies were categorized as high risk (0–3 score), moderate risk (4–6 score), or low risk (≥ 7 scores). Two investigators (M.K. and H.W.A.) independently performed the quality assessment. In case of disagreements, a final decision was reached by consulting a third investigator (A.H.).

### Data extraction

This review aimed to investigate the association between health-related outcomes of COVID-19 patients and previous influenza vaccination. By using formatted excel sheets, four authors (A.F., M.E., F.A., and K.R.) independently extracted: (1) The summary data of the eligible studies including country, study design, patients number, influenza vaccinated and non-vaccinated numbers, year of vaccination, and COVID-19 test results; (2) patient data at the start of the study including mean age, gender, and comorbidities as Hypertension, Diabetes, COPD, or asthma, renal failure, or Cardiovascular diseases; (3) outcomes including mortality rate, hospital admission, intensive Care Unit (ICU) admission, hospitalization time, ICU time, mechanical ventilation, and incidence of COVID-19 symptoms or pneumonia. A discussion with another author (H.W.A.) solved disagreements in data extraction.

### Data synthesis and statistical analysis

We analyzed the data using mean difference (MD) for continuous data and risk ratio (RR) for dichotomous data. By using the Mantel Haenszel (M–H) method, homogenous data were pooled in a fixed-effect model, while heterogeneous data were pooled using a random-effect model, with corresponding 95% confidence intervals (CI). We assessed the heterogeneity using inspection and chi-square test, and the proportion was identified by I-square tests^17^. The statistical significance was detected by *P* value < 0.05. We conducted the subgroup analysis for mortality rate to stratify patients into three groups USA, Italian, and other patients. We pooled our data using Review Manager software Version 5.4.

## Results

### Literature search and study selection

**Figure 1:**
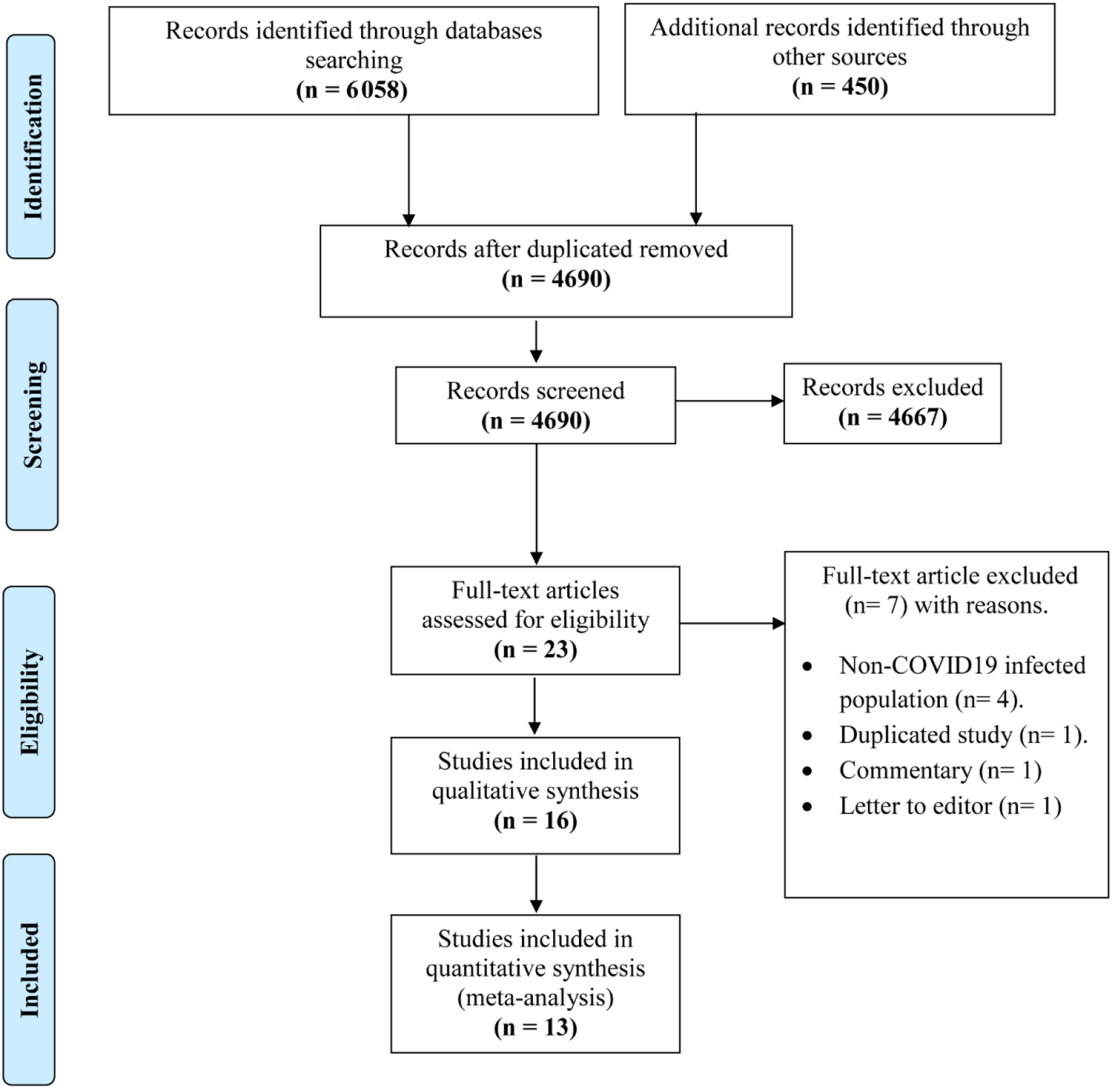
PRISMA flow chart which summarizes the literature search, and included studies.

Our search collected 6058 papers from six online databases and 450 papers from other sources, including manual search and google scholar. Of the total retrieved 6508 papers, we excluded 1818 duplications. Then 4667 papers were excluded from 4690 papers after the title and abstract screening. The remaining 23 articles were screened for the eligibility criteria. A total of 16^11–13,18–30^ papers were finally included for the final qualitative analysis, and only 13^11–13,19–22,24,25,27–30^ of them underwent quantitative analysis (Fig. 1).

### Study characteristics

Fourteen included studies were cohort studies^12,13,18–26,28–30^, while the remaining two were case-controls^11,27^. The included studies had a total sample size of 244,642 patients, of them, 191,496 COVID-19 positive patients. Most of the studies were performed in Italy^13,20,22,23,26,27^ and the USA^12,24,25,28,29^, while the others were conducted in Brazil^18^, Poland^19^, Spain^21^, Iran^11^, and England^30^. For SARS-CoV-2 infection identification, all studies used Polymerase Chain Reaction (PCR) test, however, three studies did not report the SARS-CoV-2 infection identification test used^12,22,29^. The summary of all included studies is presented in (Table 1) and patient data is presented in (Table 2).

**Table 1:** Summary of the included studies.

Study ID	Country	Design	Sample size	Age, mean (SD), or range	Influenza vaccinated from COVID-19 patient n (%)	Influenza non-vaccinated COVID-19 patient n (%)	Year of Vaccination	COVID-19 test	Influenza vaccine type
Fink et al.^18^	Brazil	Cohort	53,752*	(0–90)^$^	16,771 (31.2)	36,981 (68.8)	NR	RT-PCR	Trivalent influenza vaccine
Ragni et al.^27^	Italy	Case–control	17,608** 4885*	NR	1676 (34.3)	3209 (65.7)	2019–2020	RT-PCR	Trivalent and tetravalent influenza vaccines
Conlon et al.^12^	USA	Cohort	27,201** 1218*	47.23 (22.07)	525 (43.1)	693 (56.9)	2019–2020	NR	NR
Bozek et al.^19^	Poland	Cohort	2558** 129*	51.66 (6.24)	42 (32.6)	87 (67.4)	2020	RT-PCR	Tetravalent influenza vaccine
Wilcox et al.^30^	England	Cohort	6921*	52.4 (24.5)	2,613 (37.8)	4,308 (62.2)	2019	RT-PCR	NR
Yang et al.^29^	USA	Cohort	2005*	45.88(18.9)	214 (10.7)	1,791 (89.3)	2019	NR	NR
Candelli et al.^20^	Italy	Cohort	602*	60.6 (16.3)	150 (24.9)	452 (75.1)	NR	RT-PCR	NR
Massari et al.^13^	Italy	Cohort	115,945*	(18–85)^$^	40,117 (34.6)	75,828 (65.4)	NR	RT-PCR	NR
Umasabor-Bubu et al.^28^	USA	Cohort	588*	68.4 (14.5)	206 (35)	382 (65)	2019–2020	RT-PCR	NR
Pedote et al.^26^	Italy	Cohort	662*	55 (23.77)	190 (28.7)	472 (71.3)	2019–2020	RT-PCR	Trivalent and tetravalent influenza vaccines
Pastorino et al.^23^	Italy	Cohort	741*	66.83 (18.57)	240 (32.4)	501 (67.6)	2015–2020	RT-PCR	Trivalent and tetravalent influenza vaccine
Grico et al.^22^	Italy	Cohort	952*	59.47 (21.6)	371 (39)	581 (61)	2019–2020	NR	H1N1 Vaccination
Pawlowski et al.^25^	USA	Cohort	12,791** 963*	74.65 (9.73)	442 (45.9)	521 (54.1)	2015–2020	RT-PCR	Tetravalent and live attenuated influenza vaccines
de la Cruz Conty et al.^21^	Spain	Cohort	1150*	32.67 (6.68)	23 (2)	183 (18)	NR	RT-PCR	NR
Patwardhan et al.^24^	USA	Cohort	905*	8.42 (5.74)	439 (48.5)	466 (51.5)	NR	RT-PCR	NR
Massoudi et al.^11^	Iran	Case–control	261** 78*	39.84 (NR)	NR	NR	2019–2020	RT-PCR	Tetravalent influenza vaccine

SD, Standard deviation; RT-PCR, Reverse transcription polymerase chain reaction; (**), Total population contain positive and negative COVID-19; (*), COVID-19 positive patients; (^$^), Age in range; NR, Not reported.

**Table 2:** Baseline characteristics of patients in the included studies.

Study ID	Gender n (%)		Comorbidities n (%)															
			Hypertension n (%)		Diabetes n (%)		COPD n (%)		Asthma or COPD n (%)		Obesity n (%)		Coronary artery disease n (%)		Congestive heart failure n (%)		Current smoking n (%)	
	Male	Female	Vaccinated	Non-vaccinated	Vaccinated	Non-vaccinated	Vaccinated	Non-vaccinated	Vaccinated	Non-vaccinated	Vaccinated	Non-vaccinated	Vaccinated	Non-vaccinated	Vaccinated	Non-vaccinated	Vaccinated	Non-vaccinated
Fink et al.^18^	30,507 (56.8)	23,245 (43.2)	NR	NR	NR	NR	NR	NR	NR	NR	NR	NR	NR	NR	NR	NR	NR	NR
Ragni et al.^27^	7,898 (44.8)	9,710 (55.2)	NR	NR	NR	NR	NR	NR	NR	NR	NR	NR	NR	NR	NR	NR	NR	NR
Conlon et al.^12^	12,040 (44.3)	15,161 (55.7)	3,111 (23.9)	3,174 (22.3)	1,437 (11.1)	1,381 (9.7)	2,137 (16.4)	1,910 (13.4)	NR	NR	NR	NR	NR	NR	1199 (9.2)	958 (6.7)	581 (4.5)	1137 (8.0)
Bozek et al.^19^	1,090 (42.6)	1,468 (57.4)	NR	NR	107 (10)	124 (9)	NR	NR	178 (16)	185 (13)	NR	NR	NR	NR	NR	NR	387 (35)	459 (32)
Wilcox et al.^30^	2,801 (40.5)	4,120 (59.5)	864 (33.1)	532 (12.3)	431	170	246 (9.4)	88 (2.0)	576 (22.0)	390 (9.1)	NR	NR	277 (10.6)	91 (2.1)	144 (5.5)	52 (1.2)	974 (37.3)	1197 (27.8)
Yang et al.^29^	798 (39.8)	1,207 (60.2)	22 (10.3)	385 (21.5)	19 (8.9)	232 (13.0)	3 (1.4)	78 (4.4)	NR	NR	14 (6.5)	192 (10.7)	2 (0.9)	103 (5.8)	2 (0.9)	102 (5.7)	NR	NR
Candelli et al.^20^	390 (64.8)	215 (35.2)	96 (64.0)	152 (33.6)	26 (17.3)	40 (8.8)	22 (14.7)	24 (5.3)	NR	NR	18 (12.0)	62 (13.7)	NR	NR	NR	NR	NR	NR
Massari et al.^13^	53,121 (45.8)	62,824 (54.2)	23,837	27,225	7740	7383	3908	2906	NR	NR	NR	NR	9108	6692	NR	NR	NR	NR
Umasabor-Bubu et al.^28^	311 (52.9)	277 (47.1)	169 (82.0)	298 (78.0)	102 (49.5)	209 (54.7)	20 (9.7)	27 (7.1)	13 (6.3)	32 (8.4)	NR	NR	NR	NR	NR	NR	NR	NR
Pedote et al.^26^	317 (47.9)	345 (52.1)	NR	NR	NR	NR	NR	NR	NR	NR	NR	NR	NR	NR	NR	NR	NR	NR
Pastorino et al.^23^	452 (61)	289 (39)	NR	NR	NR	NR	NR	NR	NR	NR	NR	NR	NR	NR	NR	NR	NR	NR
Grico et al.^22^	398 (41.8)	553 (58.2)	NR	NR	NR	NR	NR	NR	NR	NR	NR	NR	NR	NR	NR	NR	NR	NR
Pawlowski et al.^25^	60,712 (44)	76,308 (56)	9216 (76.3)	9041 (74.8)	989 (8.18)	981 (8.12)	NR	NR	NR	NR	4404 (36.4)	4181 (34.6)	NR	NR	NR	NR	NR	NR
de la Cruz Conty et al.^21^	NR	206 (100)	NR	NR	NR	NR	NR	NR	4 (17.4)	8 (4.4)	NR	NR	NR	NR	NR	NR	NR	NR
Patwardhan et al.^24^	444 (49)	461 (51)	NR	NR	NR	NR	NR	NR	126 (28.70)	99 (21.24)	124 (28.25)	119 (25.32)	NR	NR	NR	NR	NR	N
Massoudi et al.^11^	141 (54.02)	120 (45.98)	NR	NR	NR	NR	NR	NR	NR	NR	NR	NR	NR	NR	NR	NR	NR	NR

*COPD* Chronic obstructive pulmonary disease, *NR* Not reported.

### The quality of the included studies

The quality assessment was assessed using the Newcastle–Ottawa scale. Based on the scale, nine^11,12,19–22,25,26,28^ out of the sixteen included studies were scored 9/9, and the other seven studies^13,18,23,24,27,29,30^ were scored 8/9. All included studies scored a low risk of bias. (Supplementary Tables 1 and 2).

### Quantitative synthesis

#### Mortality rate

**Figure 2:**
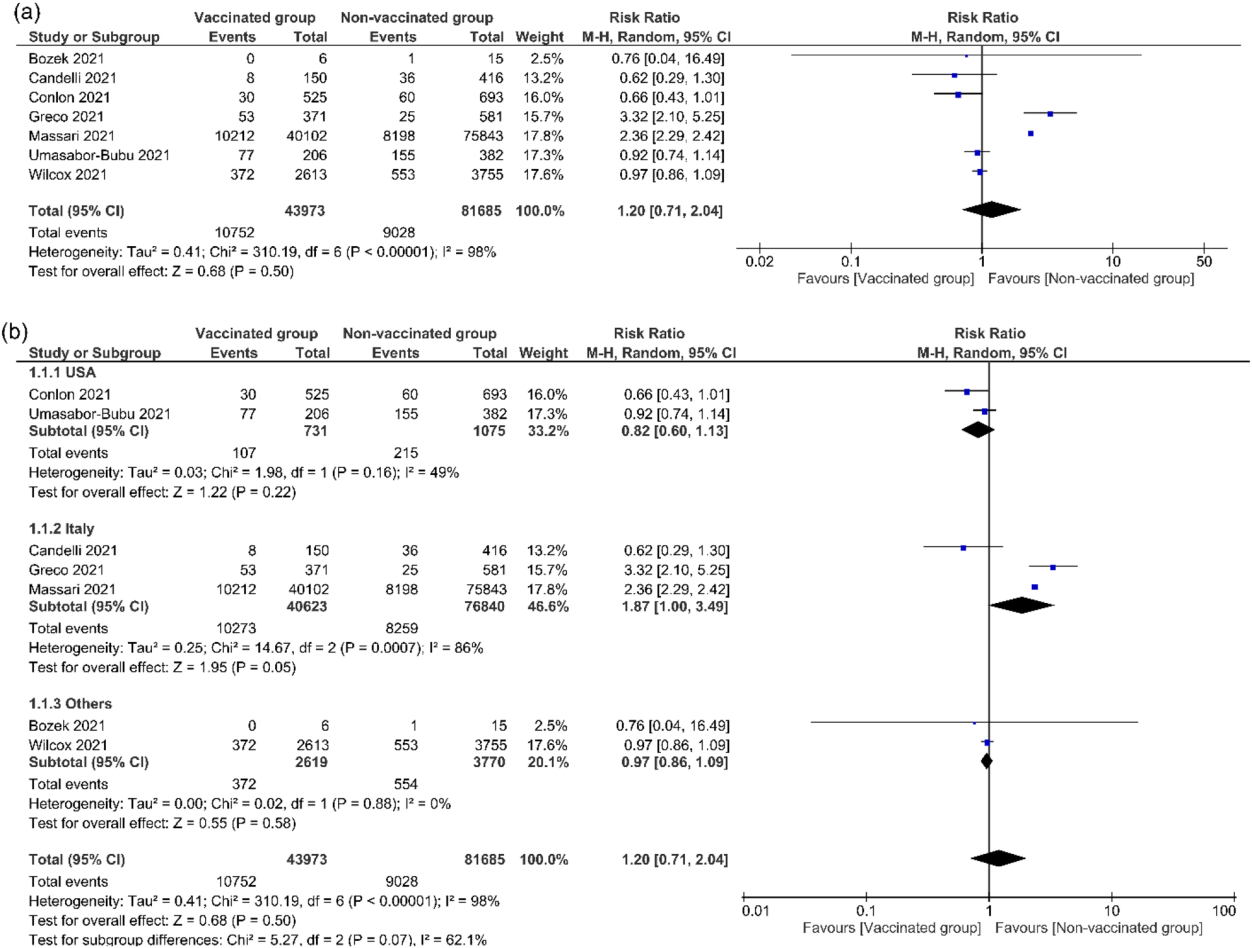
Mortality rate. This figure shows the forest plot of (**a**) Mortality rate between influenza vaccinated group and non-vaccinated group, (**b**) Subgroups of mortality rate according to countries USA, Italian, and other patients.

The pooled effect estimates of seven studies^12,13,19,20,22,28,30^ with a total sample size of 125,658 COVID-19 patients showed no significant difference between influenza vaccinated group and non-vaccinated group in the term of mortality rate (RR = 1.20, 95% CI [0.71, 2.04], *P* = 0.50; Fig. 2a). Pooled results were heterogeneous (*P* < 0.00001, I^2^ = 98%). After introducing subgroups according to the countries: USA^12,28^, Italian^13,20,22^, and other countries^19,30^, the results remain non-significant in USA and other group (RR = 0.82, 95% CI [0.60, 1.13], *P* = 0.22), (RR = 0.97, 95% CI [0.86, 1.09], *P* = 0.58), and also non-significant in the Italian group (RR = 1.87, 95% CI [1.00, 3.49], *P* = 0.05), (Fig. 2b). The heterogeneity resolved in USA, and other groups (*P* = 0.16, I^2^ = 49%), and (*P* = 0.88, I^2^ = 0%) respectively, while it remains heterogeneous in Italian group (*P* = 0.0007, I^2^ = 86%).

#### Hospital admission

**Figure 3:**
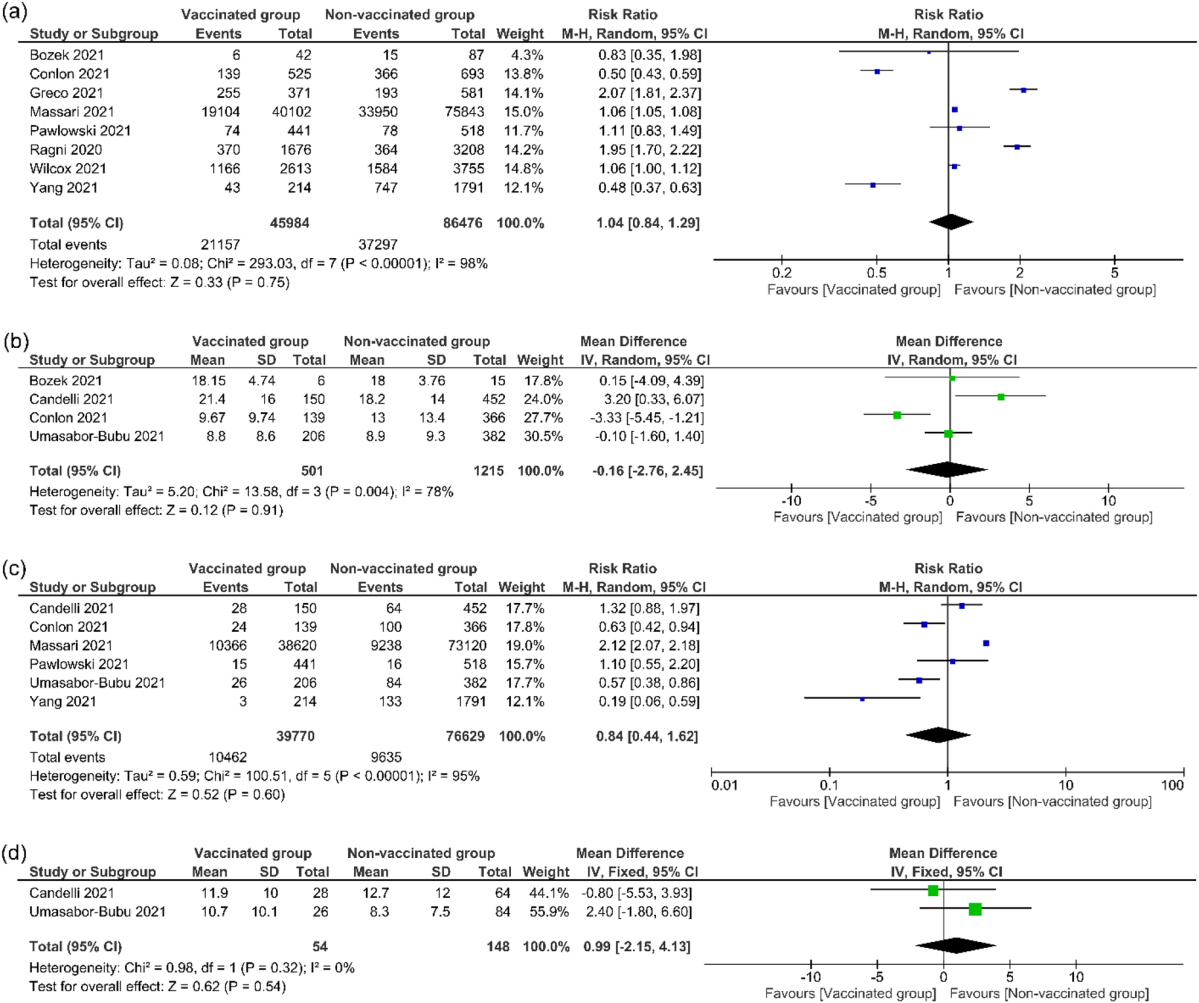
Hospital and ICU outcomes. This figure shows the forest plot of (**a**) Hospital admission, (**b**) Hospitalization time, (**c**) ICU admission, (**d**) ICU time.

The pooled effect estimates of eight studies^12,13,19,22,25,27,29,30^ (N = 132,460 COVID-19 patients) showed no significant difference between influenza vaccinated group and non-vaccinated group in the term of hospital admissions (RR = 1.04, 95% CI [0.84, 1.29], *P* = 0.75); Fig. 3a). Pooled results were heterogeneous (*P* < 0.00001, I^2^ = 98%), the heterogeneity cannot be resolved. Wilcox et al.^30^ reported the hospitalization and death, following its removal from the analysis, the results remained non-significant (RR = 1.01, 95% CI [0.71, 1.45], *P* = 0.94), and heterogeneous (*P* < 0.00001, I^2^ = 98%).

#### Hospitalization time (days)

The pooled effect estimates of four studies^12,19,20,28^ with a total sample size of 1716 COVID-19 patients indicated no significant difference between influenza vaccinated group and non-vaccinated group in the term of time staying at hospital (MD =  − 0.16 , 95% CI [− 2.76, 2.45], *P* = 0.91; Fig. 3b). Pooled results were heterogeneous (*P* = 0.004, I^2^ = 78%), and the heterogeneity could be resolved when removing Conlon et al.^12^ but the results remained non-significant (RR = 0.97, 95% CI [− 1.23, 3.17], *P* = 0.39), *P* = 0.13, I^2^ = 50%).

#### ICU admission

The pooled effect estimates of six studies^12,13,20,25,28,29^ (N = 116,399 COVID-19 patients) showed non-significant difference between influenza vaccinated group and non-vaccinated group in the term of ICU admissions (RR = 0.84, 95% CI [0.44, 1.62], *P* = 0.60); Fig. 3c. Pooled results were heterogeneous (*P* < 0.00001, I^2^ = 95%), the heterogeneity cannot be resolved. Massari et al.^13^ reported the rate of ICU admission and death composite, after removing this study from the analysis, the results remained non-significant (RR = 0.71, 95% CI [0.44, 1.16], *P* = 0.17). Pooled results showed heterogeneity (*P* = 0.002, I^2^ = 77%).

#### ICU time (days)

The pooled effect estimates of two studies^20,28^ (N = 202 COVID-19 patients admitted to the ICU) revealed no significant difference between the influenza vaccinated group and the non-vaccinated group in the term of time staying at ICU (MD = 0.99, 95% CI [− 2.15, 4.13], *P* = 0.54); Fig. 3d. Pooled results were homogenous (*P* = 0.32, I^2^ = 0%).

#### Mechanical ventilation

**Figure 4:**
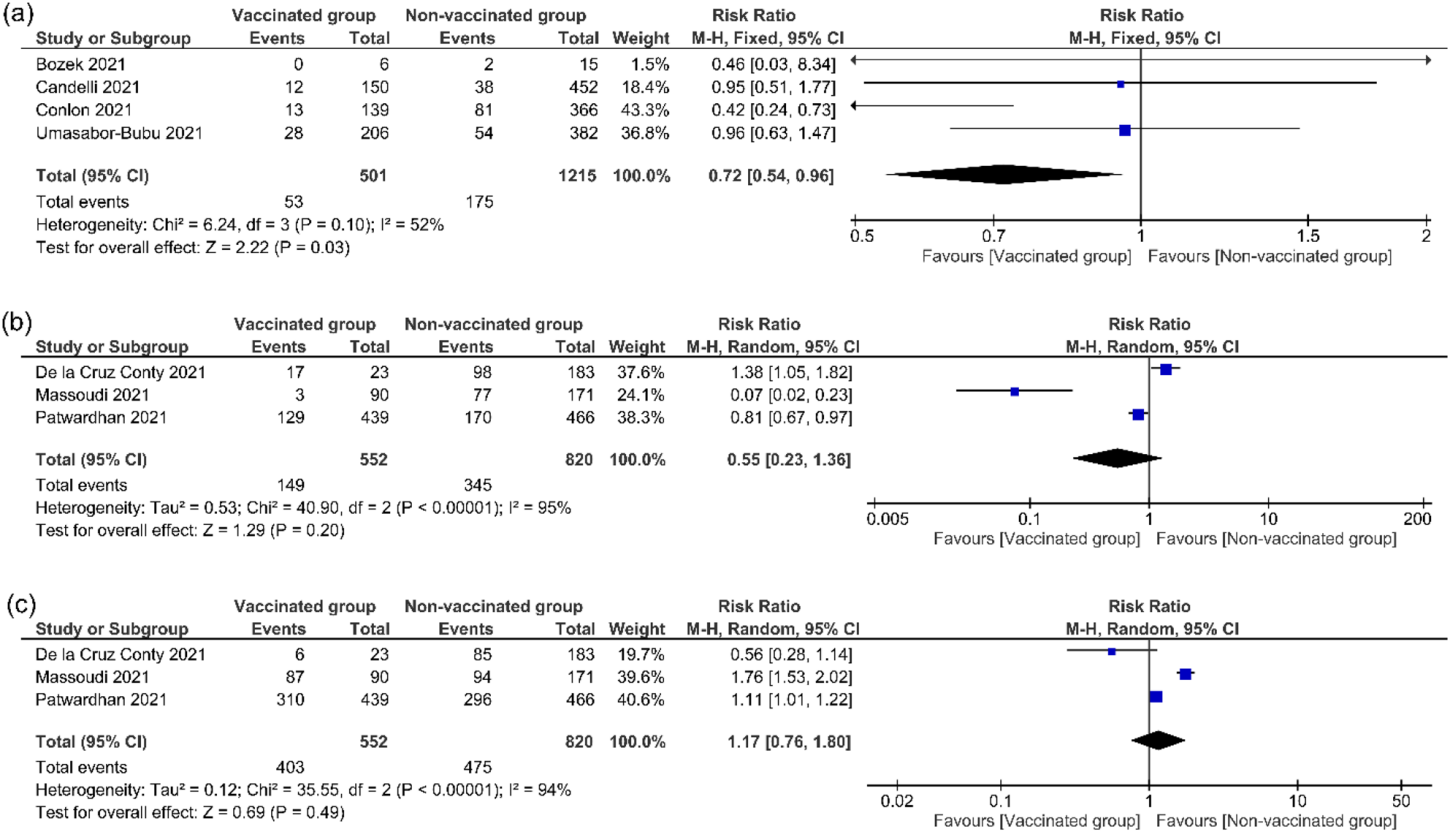
Mechanical ventilation and symptom’s appearance. This figure shows the forest plot of (**a**) mechanical ventilation, (**b**) symptomatic cases, (**c**) asymptomatic cases.

The pooled effect estimates of four^12,19,20,28^ studies of 1716 COVID-19 patients showed a significant favouring towards the influenza vaccinated group over the non-vaccinated group in the term of mechanical ventilation (RR = 0.72, 95% CI [0.54, 0.96], *P* = 0.03; Fig. 4a). Pooled results were homogenous (*P* = 0.10, I^2^ = 52%).

#### Symptoms appearing on the patient

The pooled effect estimates of three studies^11,21,24^ of 1372 COVID-19 patients indicated no significant difference between influenza vaccinated group and non-vaccinated group in the term of symptomatic and asymptomatic cases (RR = 0.55, 95% CI [0.23, 1.36], *P* = 0.20; Fig. 4b), (RR = 1.17, 95% CI [0.76, 1.80], *P* = 0.49; Fig. 4c) respectively. Pooled results were heterogeneous (*P* < 0.00001, I^2^ = 95%), (*P* < 0.00001, I^2^ = 94%) respectively, and the heterogeneity cannot be resolved.

### Qualitative synthesis

Fink et al.^18^ a retrospective cohort study report data from 53,752 Brazilian COVID-19 patients reported that patient who receive influenza vaccine were 16% lower odds of death (95% CIs [0.78, 0.90]), 7% lower odds of needing ICU treatment (95% CIs [0.87, 0.98]), and 17% lower odds of requiring respiratory support (95% CIs [0.77, 0.88]); while Pedote et al.^26^ another retrospective cohort study reported no association between influenza vaccination and mortality rate (OR = 1.6, 95% CI [0.8–3.2], *P* = 0.165), or hospitalization (OR = 1.2, 95% CI [0.7–1.9], *P* = 0.51). Also, Pastorino et al.^23^ reported the same non association results with influenza and pneumococcal vaccine.

## Discussion

Our pooled results demonstrated a significant favouring of mechanical ventilation in influenza vaccinated COVID-19 patients over the non-vaccinated ones. However, it showed no statistical differences between influenza vaccinated and non-vaccinated COVID-19 patients, in any of the mortality rates, hospital admission, hospitalization time, ICU admission, ICU time, and appearance of symptoms. Still, it is worth noting that a study by Fink et al.^18^, (N = 53,752 COVID-19 cases), could not enter the analysis but was included in the systematic review. They reported that recently influenza vaccinated patients had an average of 7% lower odds, 17% lower odds, and 16% lower odds of ICU treatment, invasive respiratory support requirement, and death, respectively. So, the results might have differed if this study was applicable to enter the analysis.

Thindwa et al.^31^ concluded that there was a relatively small magnitude of COVID-19 morbidity and mortality prevention by influenza vaccine and 23-valent pneumococcal polysaccharide vaccine, while at that time, they could not eliminate the probability of a considerable amount of prevention of COVID-19 related mortality. Our findings are similar to Wang et al.^32^ as they reported no statistical association between both groups in terms of mortality, hospitalization, or ICU in COVID-19 patients. However, they reported that patients who received the influenza vaccine showed a lower risk of SARS-CoV-2 infection. On the other hand, McIntosh et al.^33^ stated that pneumococcal and influenza vaccination could reduce SARS-CoV-2 nosocomial transmission by reducing hospitalizations for pneumonia and COVID-19 severity. They also noted that exploring co-administration of pneumococcal and influenza vaccines with a safe and effective SARS-CoV-2 vaccine is needed^33^.

Fink et al.^18^ discussed a suggestive explanation of the association between the influenza vaccine and lower risk of COVID-19 adverse outcomes. This included that the life vaccines can trigger the trained innate immunity^34,35^, and result in a recognized ‘off-target’ protection against various pathogens besides those directly targeted by the given vaccine^36,37^.

The CDC supported the safety of the influenza vaccine for pregnant women, and still, the CDC keeps on gathering data regarding this matter. The given flu shots in pregnancy aid in protecting both the pregnant woman and the baby from influenza^38^. Despite de la Cruz Conty et al. not observed a significant association between the maternal vaccination and the COVID-19 outcomes, they reported several limitations to the study. The limitations included the small sample size of influenza-vaccinated patients and their characteristics. Some of them had respiratory co-morbidities or other medical history-related factors that suggested the influenza vaccination^21^.

In children, influenza infection without vaccination increases the susceptibility to other serious illnesses^24,39,40^. So, it is crucial to consider the effect of the influenza vaccine on COVID-19 patients. One of our included studies, which involved paediatric patients, concluded that seasonal influenza and pneumococcal vaccination might be protective in symptomatic COVID-19 diseases^24^.

Globally, COVID-19 led to critical supply shortages, including ICU, hospital bed supply, hospital staff, and mechanical ventilators for affected regions^41^. Our results of significantly decreased mechanical ventilation rates in influenza vaccinated COVID-19 patients could spare efforts and lessen the burden on the healthcare system, as well as potentially provide better patients outcomes. However, this is limited because only four studies were included in the mechanical ventilation outcome, including retrospective studies. Even though our results showed no significant differences between influenza vaccinated and non-vaccinated in several COVID-19 patients' severe outcomes, this shall never deny the importance of influenza vaccination, especially during a pandemic. This can be clarified by that influenza and COVID-19 share similar symptoms and that there have been reports of their co-infections, which showed a more severe course, complications, or a fatal outcome^42,43^. In addition, COVID-19 and influenza have common high-risk groups. They are deleterious for older people and people with chronic co-morbidities, such as obesity, and residents of long-term care facilities^44,45^. Moreover, improving influenza vaccination rates will improve influenza morbidity and mortality and spare the overloaded health system during COVID-19. Eventually, this will preserve efforts and allow proper functioning without draining the resources^46^.

This study included large sample size and included studies, and each step was conducted at least twice. Moreover, all included studies had a low risk of bias. On the other hand, the limitations include the inclusion of retrospective studies which are liable to bias. Also, the results showed heterogeneity which sometimes could not be resolved. There has been a wide variety of included populations, as some studies included all COVID 19 patients, others included only hospitalized ones, one study only included pregnant patients^21^, and another one only included paediatrics population^24^. Also, there were different follow-up periods.

## Conclusion

The analysis showed a significant favor for mechanical ventilation in influenza vaccinated COVID-19 patients over the non-vaccinated ones, on the other hand, there were no significant differences between influenza vaccinated and the non-vaccinated groups among COVID-19 patients in the mortality rate, hospital admission, hospitalization time, ICU admission, ICU time, and appearance of symptoms. However, the study is limited by the heterogeneity of data and the inclusion of retrospective studies, besides that most of the included studies have not assessed viral infections other than COVID-19. Also, this should not overlook the importance of influenza vaccination, especially during the COVID-19 pandemic. Future research of high-quality randomized controlled trials is recommended to further assess the efficacy of the influenza vaccine in COVID-19 patients. The regular updating of the influenza vaccine should also be put into consideration. Other possible important confounding factors should also be taken into consideration, such as patient's health literacy, and socioeconomic status.

## Supplementary Information

Supplementary Information.
