# Supplementary material for: The effect of influenza vaccine in reducing the severity of clinical outcomes in patients with COVID-19: a systematic review and meta-analysis
Source: Sci Rep. 2022 Aug 22;12:14266. doi: 10.1038/s41598-022-18618-6 (PMC9395333; doi:10.1038/s41598-022-18618-6)
Supplement: Supplementary file 1 — Supplementary Information. [file 41598_2022_18618_MOESM1_ESM.docx]

Supplementary Table 1: Quality assessment criteria used for cohort studies through a modified version of Newcastle-Ottawa Scale.

| **Cohort studies and their assessment ratings** | | | | | | | | | | | | | | | | |  |  |
| --- | --- | --- | --- | --- | --- | --- | --- | --- | --- | --- | --- | --- | --- | --- | --- | --- | --- | --- |
|  | **Fink et al. 2020** | **Conlon et al. 2021** | **Bozek et al. 2021** | **Wilcox et al. 2021** | **Yang et al. 2021** | **Candelli et al. 2021** | **Massari et al. 2021** | **Umasabor-Bubu et al. 2021** | **Pedote et al. 2021** | **Pastorino et al. 2021** | **Greco et al. 2021** | **de la Cruz Conty M et al. 2021** | **Pawlowski et al. 2021** | | **Patwardhan et al. 2021** | | |  |
| **Selection (★★★★)** |  |  |  |  |  |  |  |  |  |  |  |  |  | |  | | |  |
| Representativeness of exposed cohort | ★ | ★ | ★ | ★ | ★ | ★ | ★ | ★ | ★ | ★ | ★ | ★ | ★ | | ★ | | |  |
| Selection of the non-exposed cohort | ★ | ★ | ★ | ★ | ★ | ★ | ★ | ★ | ★ | ★ | ★ | ★ | ★ | | ★ | | |  |
| Ascertainment of exposure | ★ | ★ | ★ | ★ | ★ | ★ | ★ | ★ | ★ | ★ | ★ | ★ | ★ | | ★ | | |  |
| Demonstration that outcome of interest was not present at start of study | ★ | ★ | ★ | ★ | ★ | ★ | ★ | ★ | ★ | ★ | ★ | ★ | ★ | | ★ | | |  |
| **Comparability (★★)** |  |  |  |  |  |  |  |  |  |  |  |  |  | |  | | |  |
| Comparability of cohorts on the basis of the design or analysis | ★☆ | ★★ | ★★ | ★☆ | ★☆ | ★★ | ★★ | ★★ | ★★ | ★★ | ★★ | ★★ | ★★ | | ★★ | | |  |
| **Outcomes (★★★)** |  |  |  |  |  |  |  |  |  |  |  |  |  | |  | | |  |
| Assessment of outcome | ★ | ★ | ★ | ★ | ★ | ★ | ★ | ★ | ★ | ★ | ★ | ★ | ★ | | ★ | | |  |
| Was follow-up long enough for outcomes to occur | ★ | ★ | ★ | ★ | ★ | ★ | ★ | ★ | ★ | ★ | ★ | ★ | ★ | | ★ | | |  |
| Adequacy of follow-up of cohorts | ★ | ★ | ★ | ★ | ★ | ★ | ☆ | ★ | ★ | ☆ | ★ | ★ | ★ | | ☆ | | |  |
| **Summary score** | **(8/9) Low** | **(9/9) Low** | **(9/9) Low** | **(8/9) Low** | **(8/9) Low** | **(9/9)**  **Low** | **(8/9) Low** | **(9/9) Low** | **(9/9) Low** | **(8/9) Low** | **(9/9) Low** | **(9/9) Low** | **(9/9) Low** | | **(8/9) Low** | | |  |
| **ID, identification; Score, (★=1, ☆=0).** | | | | | | | | | | | |  |  |  | |  | | |
| **The maximum score of each item is represented in parentheses.** | | | | | | | | | | | |  |  |  | |  | | |

Supplementary Table 2: Quality assessment criteria used for case control studies through a modified version of Newcastle-Ottawa Scale.

| **Case control studies and their assessment ratings** | | |  |
| --- | --- | --- | --- |
|  | **Ragni et al. 2020** | **Massoudi et al. 2021** | |
| **Selection (★★★★)** |  |  | |
| Is the case definition adequate? | ★ | ★ | |
| Representativeness of the cases | ★ | ★ | |
| Selection of Controls | ★ | ★ | |
| Definition of Controls | ★ | ★ | |
| **Comparability (★★)** |  |  | |
| Comparability of cases and controls on the basis of the design or analysis | ★☆ | ★★ | |
| **Exposure (★★★)** |  |  | |
| Ascertainment of exposure | ★ | ★ | |
| Same method of ascertainment for cases and controls | ★ | ★ | |
| Non-Response rate | ★ | ★ | |
| **Summary score** | **(8/9) Low** | **(9/9) Low** | |
| **ID, identification; Score, (★=1, ☆=0).** | | |  |
| **The maximum score of each item is represented in parentheses.** | | |  |
